# Editorial: Evolution, Emerging Functions and Structure of Actin-Binding Proteins

**DOI:** 10.3389/fcell.2021.819300

**Published:** 2021-12-10

**Authors:** Lei-Miao Yin, Michael Schnoor, Chang-Duk Jun

**Affiliations:** ^1^ Laboratory of Molecular Biology, Shanghai Research Institute of Acupuncture and Meridian, Shanghai University of Traditional Chinese Medicine, Shanghai, China; ^2^ Molecular Biomedicine, Center for Investigation and Advanced Studies of the National Polytechnic Institute (Cinvestav), Mexico City, Mexico; ^3^ School of Life Sciences, Gwangju Institute of Science and Technology, Gwangju, South Korea

**Keywords:** actin-binding protein (ABP), cytoskeleton, actin filament, profilin, transgelin, ezrin, post-translational modifications

## Introduction

Actin is an essential cytoskeletal protein that provides structural support for multiple cellular processes including cellular motility, division and contractility (Pollard and Goldman, 2018). Actin filaments exist in association with binding partners, mainly actin-binding proteins (ABPs) that together control the many functions of the actin cytoskeleton (Lehman and Maeda, 2020). ABPs can bind actin monomers, polymers or both and more than 160 ABPs have been identified to date (Winder and Ayscough, 2005). However, classification of ABPs is difficult because actin binding is a common process, but can result in a plethora of different functional outcomes. Therefore, attempts to classify ABPs has left many “orphans” that do not fit into families (dos Remedios et al., 2003). Generally, ABPs can be divided into two broad categories, depending on their effect on actin filament dynamics (Stehn et al., 2006). The first category of ABPs regulates the G-actin/F-actin turnover thus controlling cytoskeletal responses to external stimuli. This category includes Arp2/3, ADF/cofilin, profilin, gelsolin, etc (Stehn et al., 2006). The second general category of ABPs helps actin filamnets to form higher order structures, such as actin filaments meshworks or bundles. This category includes tropomyosin, caldesmon, filamin, dystrophin, among others (Stehn et al., 2006).

**FIGURE 1:**
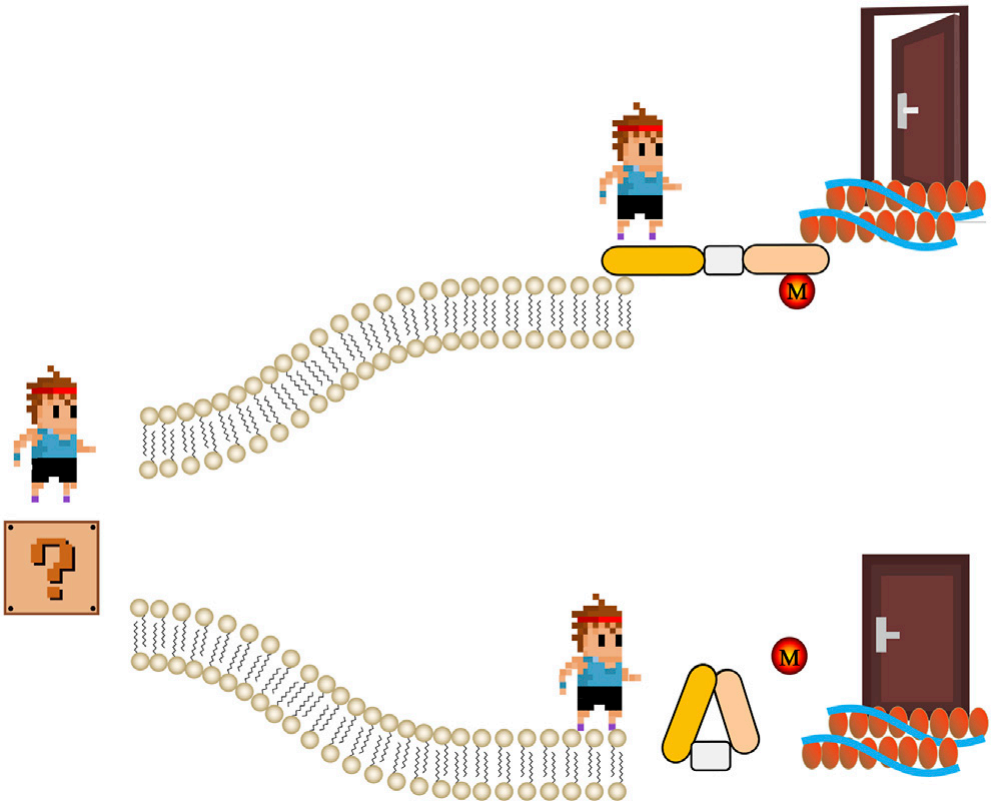
The schematic of regulation of actin-binding proteins by post-translational modifications in signaling. Extracellular signal (represent by a cartoon boy standing in a question mark) meets two roads made by phospholipid bilayer. If actin-binding proteins are active (usually regulated by post-translational modifications, M), signal can go through and get to cytoskeleton. Therefore, the door that representing biological function of extracellular signal is open. On the contrary, if the actin-binding proteins are not active, cartoon boy stops in the other road.

ABPs also link actin filaments to the plasma membrane, thus facilitating outside-in and inside-out signaling (Figure 1). For example, dystrophin anchors the actin cytoskeleton to membrane glycoproteins such as β-dystroglycans that binds to the extracellular matrix protein laminin. Dystrophin mutations resulting in truncated dystrophin proteins unable to bind to the membrane cause progressive muscle degeneration and atrophy (Xu L. et al., 2021). The unconventional myosins Myo1e and Myo1f are motor ABPs that can connect the actin cytoskeleton with the plasma membrane via transmembrane Fcγ receptors (FcR) in macrophages to control membrane tension during FcR-mediated phagocytosis (Barger et al., 2019). Absence of both myosins significantly impairs actin-dependent phagocytic cup formation and clearance of pathogens. Myo1e is also important for actin polymerization and integrin clustering in neutrophils during extravasation (Vadillo et al., 2019). These are only a few examples highlighting the important cell biological functions of ABPs in different pathophysiological contexts. With newly identified biochemical and biological properties of ABPs, they have been considered as important targets in treating diseases (Yin et al., 2018b; Vadillo et al., 2019; Yin et al., 2019). This special issue contains 31 original and review papers, that discuss the recent advances in our knowledge about ABPs and their various biological processes. These studies also highlight new avenues for future ABPs research.

Profilin and the actin-related protein 2/3 (Arp2/3) complex are key regulators of actin polymerization and branched actin networks, respectively. Pandey and Chaudhary review the evolution of the profilin gene family in plants, discussing that profilins play important roles in both cytoskeleton maintenance and plant development (Pandey and Chaudhary). Murk et al. discuss the different expression patterns and cellular functions of profilin isoforms, and their relevance in neurological diseases (Murk et al.). Ren et al. show that profilin-1 and protein tyrosine phosphatase receptor S are potential suppressors in the recurrence and metastasis of malignant peripheral nerve sheath tumors (Ren et al.). Dimchev et al. show the direct and indirect effects of acute Arp2/3 complex removal on actin cytoskeleton regulation (Dimchev et al.). Chanez-Paredes et al. show that loss of the Arp2/3 inhibitory protein arpin triggers dysfunction of the intestinal epithelial barrier during inflammation and that low arpin levels may be a novel hallmark of acute colitis (Chánez-Paredes et al.). Opposing profilin and Arp2/3, actin depolymerizing factor (ADF)/cofilin can depolymerize actin filaments. Ben Zablah et al. show that ADF/cofilin regulates synaptic structure and functions in the brain, and may therefore be an important target in Alzheimer’s Disease (Zablah et al.). Xu et al. show that changes in cofilin-1 expression appear at the same time as gait imbalance suggesting that it may affect motor cortex function (Xu et al.). Xu et al. review the structural features, phosphorylation patterns and functions of cofilin in regulating cancer metastasis and apoptosis, highlighting that cofilin may be a therapeutic target for treating cancers (Xu et al.).

Three transgelin isoforms exist that are named for their potential to induce actin gelation (Yin et al., 2019). Vakaloglou et al. show that *drosophila* transgelin proteins Mp20 and CG5023 modulate muscle function, whereas another transgelin isoform in *drosophila*, Chd64, has distinct roles in epithelial, neuronal, and endodermal tissues based on their distinct tissue expression (Vakaloglou et al.). Liu et al. highlight the potential role of transgelin-1 and transgelin-2 as biomarkers and therapeutic targets for metastasis of colorectal cancer (Liu et al.). Analysis of transgelin-3 gene structure, expression regulation and potential biological functions in neurological disorders is described by Wu et al. Transgelins have a calponin homology (CH) domain, one of the most common modules in various ABPs (Yin et al., 2018b). The structural features, interactome and related diseases of the CH domain are reviewed by Yin et al. Riviere et al. summarize the functions, genetic analyses and expression regulation of leucine-rich repeat and CH domain-containing (LRCH) proteins in leukocyte biology (Rivière et al.).

Gupta et al. review the intracellular distribution, structure and functions of actin isoforms and ABPs in *trypanosomatids* (Gupta et al.). Functions of filamin A and the adhesion and motility mechanisms are summarized by (Lamsoul et al.). The relationship of plastin-3 with osteogenesis imperfecta, functions of plastin-3 and its regulation of calcium signaling have been summarized by (Schwebach et al.). Gene transcription, isoform structure, expression and phosphorylation regulation of caldesmon have been reviewed by Yao et al. Rust et al. summarize the molecular, developmental and physiological functions of cyclase-associated protein, and discuss its cellular functions and role in human diseases (Rust et al.). Ji et al., 2020 review the role of cortactin in epithelial-mesenchymal transition and cancer development (Ji et al.). Park et al. show that α-actinin-4 is involved in androgen-independent prostate cancer transition (Park et al.). Mun et al. demonstrate that the mitochondrial ABP EF-hand domain-containing protein 1 contributes to mitochondrial morphology and energy synthesis (Mun et al.). Gao et al. provide evidence that CD47 contributes to lipid nephrotoxicity and that CD47-targeted therapy protects cells from epithelial-mesenchymal transition and inflammation (Gao et al.). Hou et al. show that coactosin promotes the assembly of protrusive actin filament arrays at the leading edge for growth cone motility (Hou et al.). Flightless I is a member of the gelsolin family of ABPs that is a potential target in proliferation regulation (Strudwick and Cowin). Ren et al. review the relationships between programmed cell death and the actin machinery, and discuss new therapeutic strategies for treating aging or cancers (Ren et al.). Lechuga et al. demonstrate that β-actin is an essential regulator of intestinal epithelial barrier integrity during mucosal injury and inflammation (Lechuga et al.). Dai et al. show that the compound echinacoside inhibits the phosphorylation of the double-stranded RNA-activated protein kinase (PKR)-like endoplasmic reticulum kinase, and promotes F-actin accumulation leading to reduced communication of endoplasmic reticulum and plasma membrane (Dai et al.).

Regulation of ABPs by post-translational modifications such as oxidation and phosphorylation is pivotal to the rapid responsiveness of cells to their environment (Figure 1). Balta et al. focus on the redox regulation of the actin cytoskeleton and pathophysiological consequences (Balta et al.). Ezrin-mediated membrane-cytoskeleton interactions are also controlled by phosphorylation (Yin et al., 2018a; Yin and Schnoor, 2021). Song et al. review the structures, protein interactions, and oncogenic roles of ezrin; and discuss applications of new compounds targeting ezrin and ezrin posttranslational modifications in basic and clinical studies (Song et al.).

New technologies and new methods for the study of ABP biology have recently been developed. Jung et al. review the direct visualization of ABPs and actin using novel electron microscopy, super resolution microscopy and correlative light and electron microscopy techniques (Jung et al.). Shigene et al. suggest image translation via artificial intelligence, i.e. using the convolutional network to predict the localization of cytoskeletal proteins (Shigene et al.).

## Conclusion and Future Perspective

With the help of ABPs, actin filaments can be assembled, elongated, branched, disassembled and formed into dynamic networks as response to extracellular stimuli (Tang, 2018). As ABPs are essential for actin filament dynamics, they are closely related to various human diseases. Several drugs targeting ABPs (e.g. dystrophin, tropomyosin, troponin, etc) have been developed to treat disorders related to actin or myosin dysfunctions, many of which have been approved by the FDA. For example, the antisense oligonucleotide Vyondys 53 has been approved for the treatment of Duchenne muscular dystrophy in patients with a confirmed mutation amenable to exon 53 skipping (Frank et al., 2020). Levosimendan targets troponin C for treating low cardiac output syndrome after cardiac surgery is being tested in phase III trials (Pollesello et al., 2019). These are only two recent examples and numerous other clinical trials testing new ABP-targeting compounds have been launched. Despite their potential, there is certainly more room for testing ABPs as targets in different diseases because they are ubiquitously expressed and involved in many different crucial cellular functions. In this respect, this special issue provides timely research and scholar overviews that shed new light on ABP functions in health and disease. We hope our efforts will help researchers to acquire a better understanding of ABPs.
